# P06-06 Co-development of the national monitoring system for the joy of motion, physical activity and motor skills for pre-school-aged children in Finland

**DOI:** 10.1093/eurpub/ckac095.091

**Published:** 2022-08-29

**Authors:** Anette Mehtälä, Arja Sääkslahti, Tuija Tammelin

**Affiliations:** 1 LIKES Research Centre for Physical Activity and Health, Jyväskylä, Finland; 2 Faculty of Sport and Health Sciences, University of Jyväskylä, Jyväskylä, Finland

**Keywords:** co-creation

## Abstract

In recent years, we have got more information on physical activity and motor skills of preschool-aged children in Finland. However, national monitoring system for this age groups is still lacking in Finland like in many other European countries too. This presentation describes the process and recent results of an on-going research and development project JOYPAM - monitoring the joy of motion, physical activity and motor skills for pre-school-aged children. Aim of the project is to co-create, test and recommend a national level monitoring system by the end of 2020.

